# Ilizarov Fixation Method of Tibia Plateau Fractures: A Prospective Observational Study

**DOI:** 10.7759/cureus.11277

**Published:** 2020-10-31

**Authors:** Shahzaib R Baloch, Mohammad S Rafi, Javeria Junaid, Mohammad Shah, Faateh Siddiq, Syed Ata-ur-Rahman, Zardana Zohaib

**Affiliations:** 1 Orthopaedics, Dr. Ziauddin University Hospital, Karachi, PAK; 2 Biochemistry, Mekran Medical College, Turbat, PAK

**Keywords:** schatzker tibia plateau classification, ilizarov, external ring fixator, tibia plateau fracture, knee injury and osteoarthritis outcome score, gastilo anderson classification

## Abstract

Background: Tibia plateau fractures are most commonly managed with open reduction and internal fixation (ORIF) technique, external fixation via minimally invasive technique are an excellent alternative. The aim of this study was to assess the results of tibia plateau fractures by using the Ilizarov external fixator. The analysis was done both clinically and radiologically.

Methodology: Some 72 patients with isolated tibia plateau fractures were brought to ED and clinics and assessed. The fractures were classified according to Schatzker Tibia Plateau Fracture classification, only Schatzker type III to VI were included using conventional X-rays. All patients for their tibia plateau fractures underwent surgical correction using Ilizarov technique with full weight bearing and knee range of motion allowed the next day. Patient follow-up up to one year was done. KOOS and self-appraisal were used to evaluate the knee pain and function.

Results: All the fractures healed with 67 patients achieving a range of motion better than 0-100º. KOOS score shows that patients who had worse fracture patterns (Schatzker-V and VI) have worse global scores (p=0.002); still when managed with an Ilizarov it has been seen that these patients are able to maintain a moderately pain free knee (p=0.013) other aspects of the KOOS. Patients with higher BMI showed to have worsening fracture patterns with 20 out of the 30 overweight patients suffering Schatzker-V (66.67%). We experienced an extremely low rate of pin tract infections with only two debridements required.

Conclusion: Ilizarov external fixation method is a valuable alternative treatment with excellent clinical outcomes and early mobilization.

## Introduction

Fractures of the tibia plateau are a relatively common occurrence when we speak about lower limb fractures, as they contribute to approximately 1% of all fractures [1-3]. The anatomical significance in such cases is due to the fact that the articular surface is involved and these fractures are usually a resultant of a high impact injury [4]. The mechanism of the injury contributes to a high rate of complications [5] and it also tends to cause severe soft tissue damage [4].

The nature of these fractures demands active intervention especially due to the nature of the fracture involving a joint. A clinician’s goal is to provide a solution or a form of treatment that restores the normal anatomy, by providing optimal stability and mobility of the joint along with pain relief and causing minimal postoperative complications [6-7]. Historically the recommended treatment for tibia plateau fractures, specifically Schatzker Type I-IV, has been open reduction and internal fixation (ORIF) using plates and screws. Advanced forms of the fracture pertaining to Schatzker Type V-VI have also previously been managed by this modality but recent use of the Ilizarov technique, using a circular fixator, has opened room for debate in the management of these fractures [8].

The Ilizarov offers a minimally invasive approach where multiple small stab incisions are required for adequate intervention with minimum soft tissue destruction and blood loss [9-10], whereas previously used methods left a much larger scar [11]. Other advantages of this technique benefit the mechanics, as it provides good stability and accurate alignment can be achieved as adjustments during and after surgery are possible. Most importantly, the Ilizarov is remarkably beneficial for the mobility of the patient as it permits early weight bearing [12-13]. This is a good prognostic factor for patients postprocedure.

Medical literature is devoid of an extensive view regarding the use of Ilizarov in these fracture, over previously adopted techniques. An article by Watson and Coufal is one of the few pieces that provide evidence that the Ilizarov gives excellent results [14]. The aim of our research is to contribute towards determining the best treatment modality with limited resources for the management of tibia plateau fractures. We have gathered retrospective data from patients we have treated in our hospital with the Ilizarov and shown they have fared well postoperatively. We aim to pose an argument for the use of this technique against the previously used management options, while also giving focused insight specific to patients in the South Asian region.

## Materials and methods

**Figure 1:**
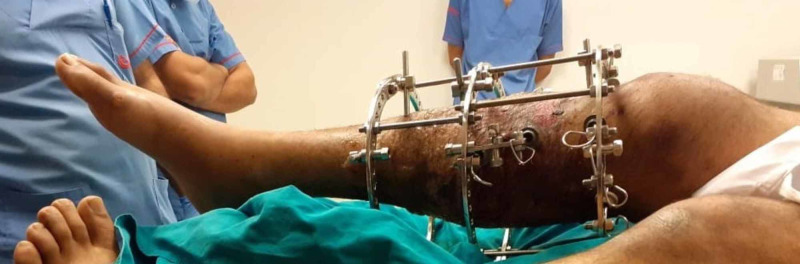
Ilizarov frame.

**Figure 2:**
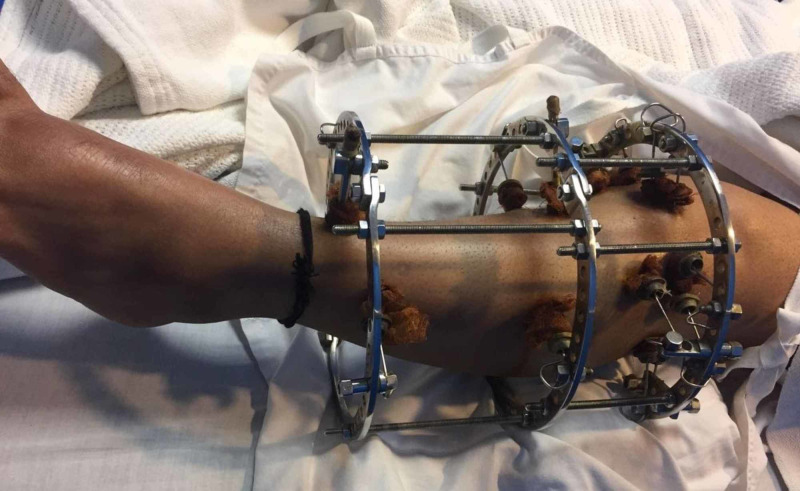
Image of one of our patients on outpatient visits with Ilizarov frame.

**Figure 3:**
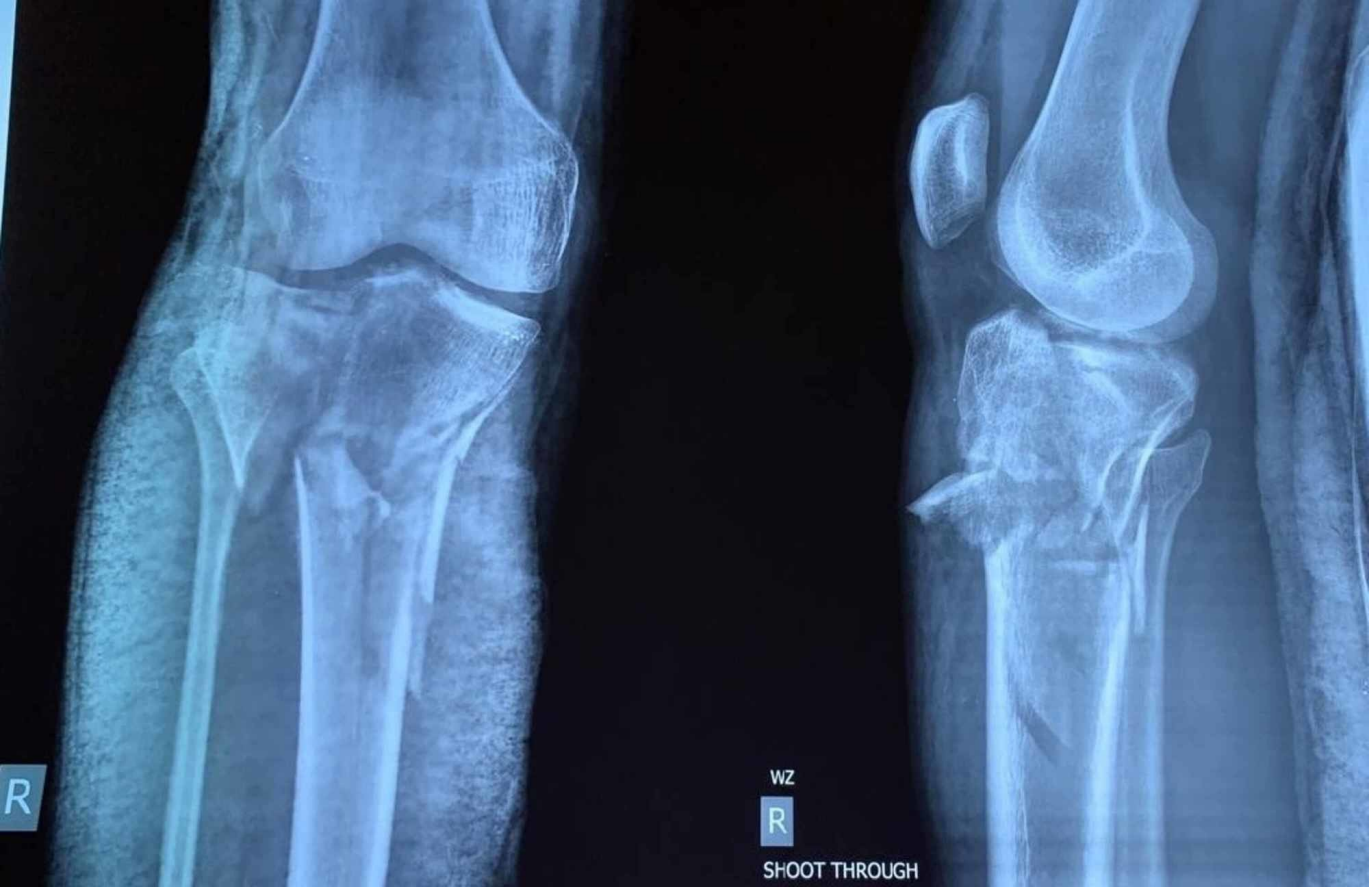
Preoperative X-ray AP/lateral views of the right knee joint. AP, anteroposterior

**Figure 4:**
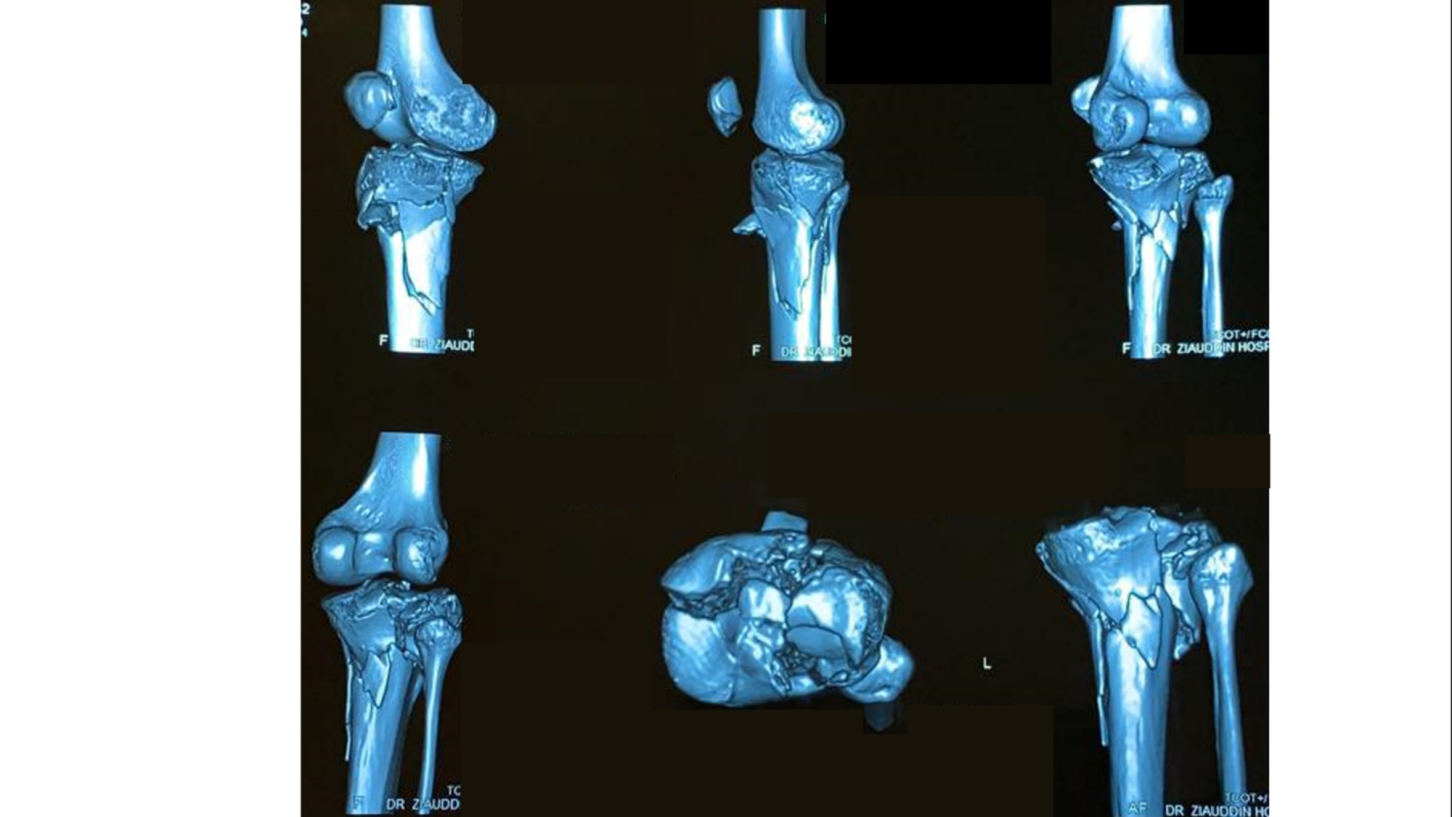
Preoperative CT scan image of the left knee joint of one of our patients.

**Figure 5:**
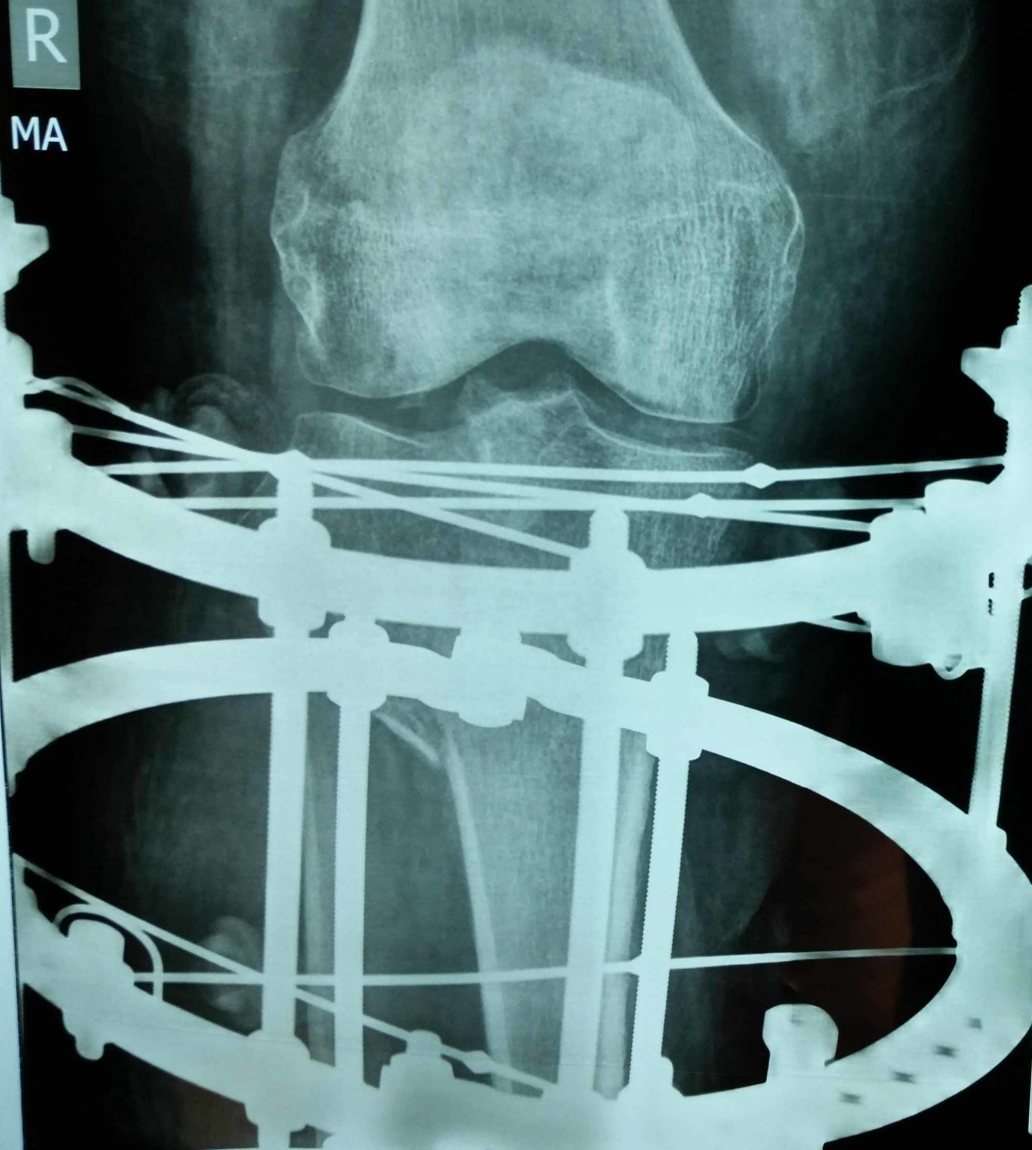
Postoperative X-ray of the knee joint with Ilizarov.

Between 2015 and 2018, 72 patients, who suffered tibia plateau fractures were admitted at our institute either via Emergency & Accident or outpatients department were included in the study. All the 72 patients were treated by the Ilizarov fixator external ring fixators using two full rings and one 5/8 ring (Figures 1-2). Out of these 50 were males and 22 were females; the mode injury was assessed but was not the reason for excluding any patients. Patients with neurovascular compromise or Gustilo Anderson type III-C were excluded. Patients who were included had presented to us with unicondylar and bicondylar fractures of the tibia plateau and were classified using the Schatzker Tibial Plateau Fractures classification system. Initial X-rays both anteroposterior (AP) and lateral views (Figure 3) were taken and for complex fractures CT scan (Figure 4) imaging was also done. These patient’s level of function, clinical and radiological findings, and the patient opinions were assessed at an interval of at least 12 months postoperatively. Some 50 (69.4%) males and 22 (30.6%) females who met the inclusion criteria for the study, had a mean and standard deviation for age of 39 ± 25 years. Some 18 patients were front seat drivers or passengers, nine were pedestrians struck by motor vehicles and 39 were motor-cycle riders, four had been injured by falls from a height and two had a crush injury caused by a collapsed wall. Eleven (15.3%) of the fractures were open. Out of the total 72, 19 (26.4%) were found to be Schatzker (1987) Type-V and 5 (6.9%) Type-VI fractures; 14 (19.4%) amongst these were comminuted fractures. Twenty patients (27.8%) were found to have had a complex knee trauma which is the name given to injuries that include more than one of the functional compartments of the knee [11]. The functional compartments of the knee include the soft-tissue envelope, the ligamentous stabilizers, and the bony structures of the distal femur and the proximal tibia. Figure 5 shows the postoperative radiograph of one of our tibial plateau fracture patients showing pins arrangement after fracture reduction.

 

 

 

 

**Figure 6:**
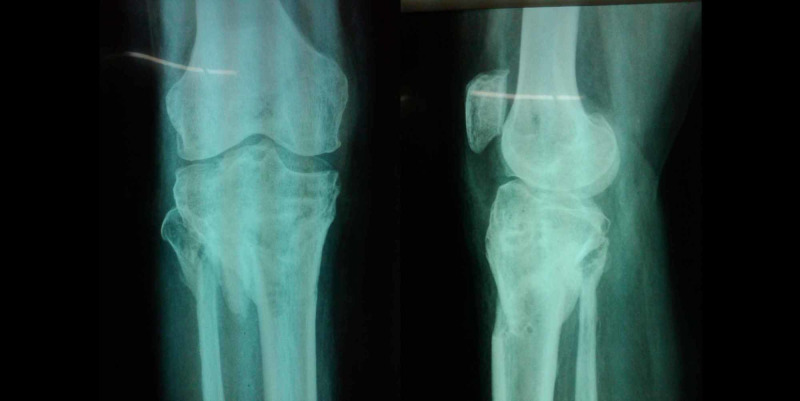
Knee joint X-rays postremoval showing healed fracture.

**Figure 7:**
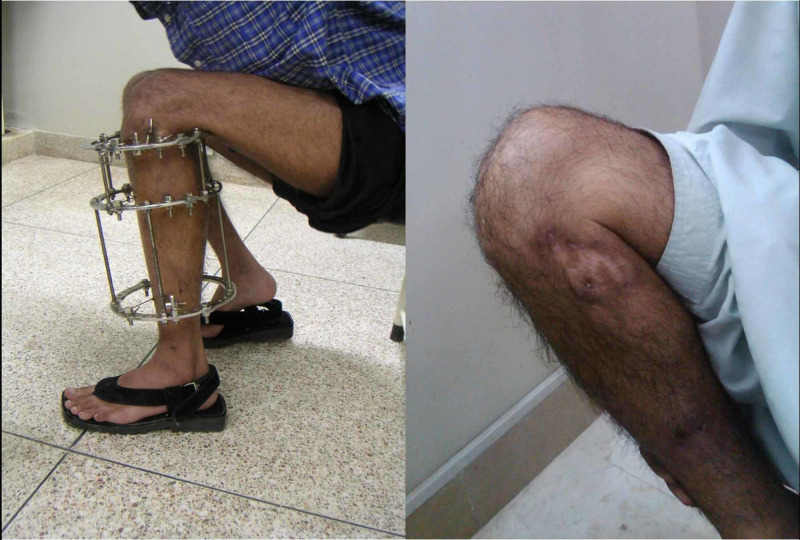
Image demonstrating range of motion of a patient with and without Ilizarov fixator.

On follow-up only 31(43.1%) patients were available for interview after one year. Out of these 26 males and five females, it was seen that all the mechanisms of injury were motor vehicle accidents. There were 15 (48.4%) Schatzker-V and 10 (32.3%) Schatzker-VI injuries. Of these open fractures of grade-IIIA (Gustilo and Anderson) were encountered in six patients. Eight patients had severe closed soft tissue injuries, including internal de-gloving and there were three cases of compartment syndrome. The diagnosis of compartment syndrome was based on clinical examination and these patients had to undergo an emergency fasciotomy followed by reduction of the fracture and application of hybrid fixators. Figure 6, demonstrating an image of a radiograph of knee joint (AP/lateral views) of one of our patients after removal of Ilizarov fixator and healed fracture can be seen. Range of motion of knee joint with Ilizarov fixator and after removal of Ilizarov is shown in Figure 7.

## Results

**Figure 8:**
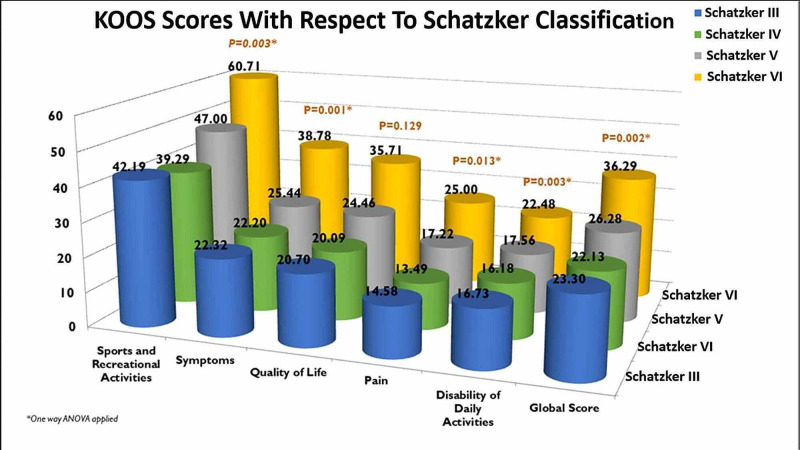
KOOS scores with respect to Schatzker classification.

Our results with the KOOS score show that patients who had worse fracture patterns (Schatzker V and VI) have worse global scores (p=0.002) as expected, still when managed with an Ilizarov it has been seen that these patients are able to maintain a moderately pain free knee (p=0.013); other aspects of the KOOS are shown in Figure 8.

**Figure 9:**
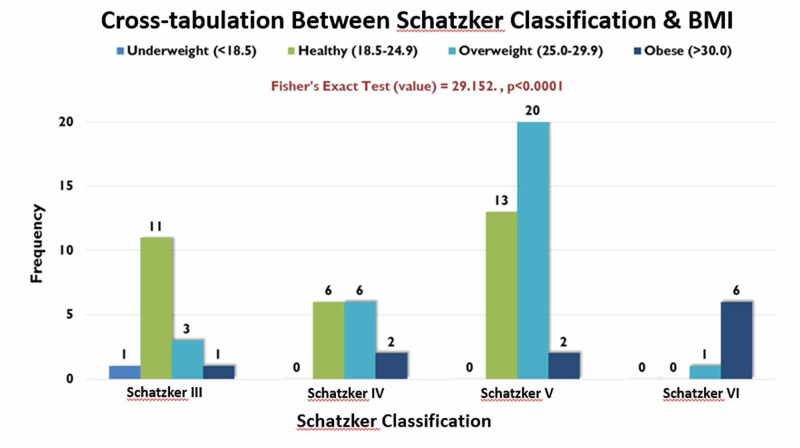
Cross-tabulation between Schatzker classification and BMI. BMI, body mass index

Patients with higher BMI showed to have worsening fracture patterns with 20 patients out of the 30 overweight patients suffering Schatzker V (66.67%) when interpreted using a Fishers exact test (p < 0.001); this can be seen in Figure 9.

**Figure 10:**
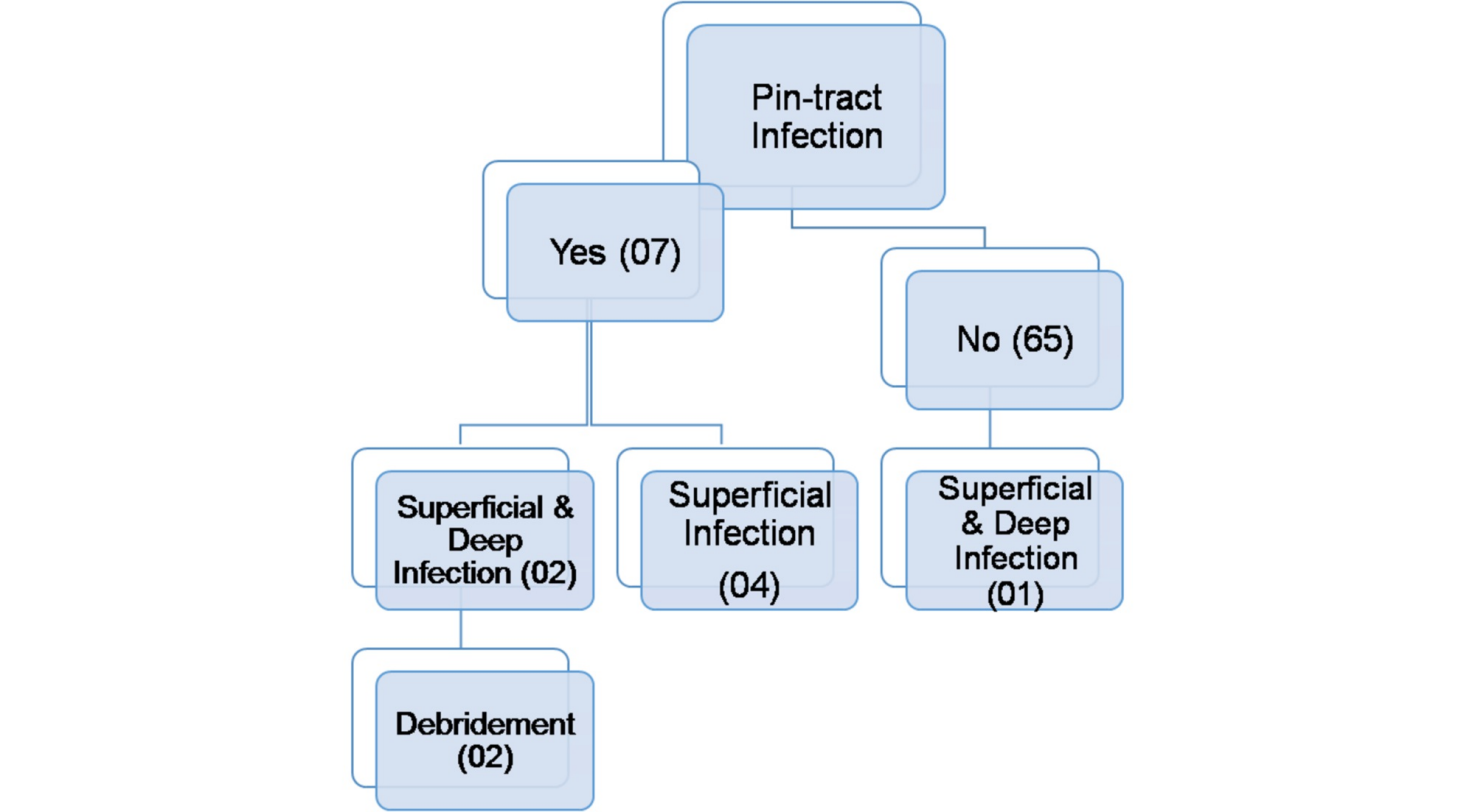
Tree diagram of infection tract.

We experienced an extremely low rate of pin tract infections with only two debridements required, during the intervention as seen in Figure 10, as we emphasized on patient education and taught everyone how to do pin tract dressings themselves.

**Figure 11:**
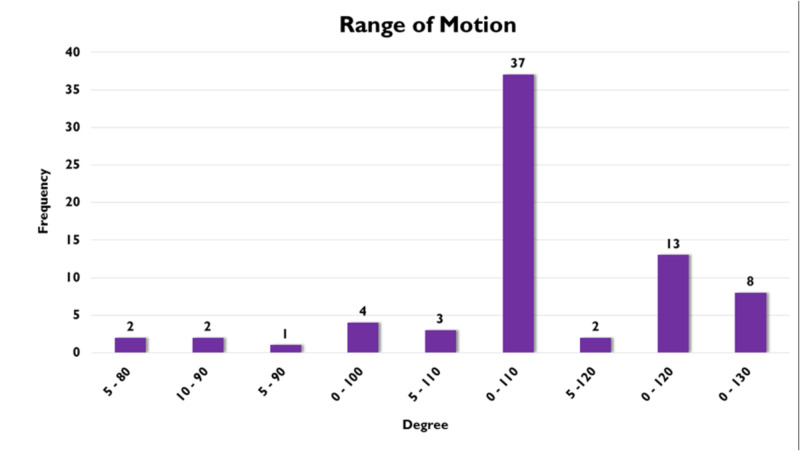
Knee range of motion one year postsurgery.

Most patients on the study were able to achieve adequate range of motion, with no fixed flexion contractures and bending greater than 90 degrees (which is required for sitting and getting up from sitting position) as seen in Figure 11.

 

 

 

## Discussion

It is clearly evident from the results of our study that a vast majority of tibial plateau fractures (Schatzker II-VI) treated with an external fixator achieved early range of motion upto 110◦ with early weight bearing without endangering the stability of the fracture fragments or impeding the healing process as published by Ramos et al. [15]; these findings favor this treatment modality in such injuries.

Another important finding of this study is the positive correlation of increasing BMI with worsening fracture type: obese patients mostly sustaining the worst type of fracture i.e. Schatzker VI and on the far end, healthy patients dominating the Schatzker III injury group; hence proving that greater the patient’s BMI, greater the possibility of a higher Schatzker type injury anticipated.

One of the highlighted superiority of Illizarov over ORIF is low rate of infection, also observed in this study. Out of 72 patients, 65 had an infection free course of treatment whereas only seven patients acquired pin tract infection either superficial or deep. Similar outcomes were noted by Catagni et al. [16]. Parameswaran et al showed an incidence of 0% in deep infection in 59 patients with Schatzker V-VI, managed with Illizarov fixation [17]. Similarly Colman et al. in their study stated ring fixators to have the lowest incidence of infections when comparing various external fixation devices [18], they treated 30 patients using Illizarov technique and registered most of the infections as “easy-to-treat superficial pin-site” infections. Apart from being a closed method, lesser surgical time required for tibial plateau management via Illizarov technique is an added benefit in comparison of more time consuming ORIF of tibial plateau [18]. Better gait pattern was seen in patients who were treated with ring fixators and also in another which showed lower complication by Elsoe et al. [19-20].

Debnath et al. [21], Gill and Raza [22], Zhao et al. [23], and Nesari et al. [24] believe that the Ilizarov technique is better for the management of complex tibial plateau fractures, which have extensive comminution with compromise of soft tissue and should be preferred over other methods. Whereas Tahir et al. [25], Pineda et al. [26], Metcalfe et al. [27], and Boutefnouchet et al. [28] believe that mode of fixation (ORIF or Ilizarov ring fixator) do not make any difference in the outcome.

The KOOS tool was utilized to assess the impact of tibial plateau fracture after fixation via Illizarov technique on major aspects of the patient’s life. KOOS is a patient-reported outcome measurement instrument used to subjectively assess the patient’s knee functionality and associated limitations including sports and recreational activities, symptoms, quality of life, pain, and disability of daily activities. This 42-item-based questionnaire gages both short-term and long-term consequences of knee injury and the repercussions of osteoarthritis in the long run. The sub-classes include: KOOS pain, KOOS symptoms, and function in daily living (KOOS ADL), function in sport and recreation (KOOS-sport/rec), and knee-related quality of life (KOOS QOL). The average scores for all these elements were calculated separately and a combined average score exhibited an increasing trend of scores paralleling the fracture type with highest scores for Schatzker VI; however, a slight decrement in scores from Type III to IV is an odd finding. Hence we can draw out a conclusion that the patients tend to have a greater level of restriction and decreased functionality in the aforementioned domains as the fracture class increases; on the other hand, down sloping off KOOS from III to IV is an interesting finding.

## Conclusions

For tibial plateau fractures, Ilizarov external fixation method is a valuable alternative treatment with excellent clinical outcomes, with early mobilization. The better the fracture pattern with less comminution results in better postoperative functional outcome.
